# Genomic analysis of Ugandan and Rwandan chicken ecotypes using a 600 k genotyping array

**DOI:** 10.1186/s12864-016-2711-5

**Published:** 2016-05-26

**Authors:** D. S. Fleming, J. E. Koltes, A. D. Markey, C. J. Schmidt, C. M. Ashwell, M. F. Rothschild, M. E. Persia, J. M. Reecy, S. J. Lamont

**Affiliations:** Iowa State University, Ames, IA USA; University of Delaware, Newark, DE USA; North Carolina State University, Raleigh, NC USA; Virginia Polytechnic University, Blacksburg, VA USA; University of Arkansas, Fayetteville, AR USA

**Keywords:** Homozygosity, Selection signatures, Stress response

## Abstract

**Background:**

Indigenous populations of animals have developed unique adaptations to their local environments, which may include factors such as response to thermal stress, drought, pathogens and suboptimal nutrition. The survival and subsequent evolution within these local environments can be the result of both natural and artificial selection driving the acquisition of favorable traits, which over time leave genomic signatures in a population. This study’s goals are to characterize genomic diversity and identify selection signatures in chickens from equatorial Africa to identify genomic regions that may confer adaptive advantages of these ecotypes to their environments.

**Results:**

Indigenous chickens from Uganda (*n* = 72) and Rwanda (*n* = 100), plus Kuroilers (*n* = 24, an Indian breed imported to Africa), were genotyped using the Axiom® 600 k Chicken Genotyping Array. Indigenous ecotypes were defined based upon location of sampling within Africa. The results revealed the presence of admixture among the Ugandan, Rwandan, and Kuroiler populations. Genes within runs of homozygosity consensus regions are linked to gene ontology (GO) terms related to lipid metabolism, immune functions and stress-mediated responses (*FDR < 0.15*). The genes within regions of signatures of selection are enriched for GO terms related to health and oxidative stress processes. Key genes in these regions had anti-oxidant, apoptosis, and inflammation functions.

**Conclusions:**

The study suggests that these populations have alleles under selective pressure from their environment, which may aid in adaptation to harsh environments. The correspondence in gene ontology terms connected to stress-mediated processes across the populations could be related to the similarity of environments or an artifact of the detected admixture.

**Electronic supplementary material:**

The online version of this article (doi:10.1186/s12864-016-2711-5) contains supplementary material, which is available to authorized users.

## Background

In nature, environmental stressors can influence the phenotypic characteristics that individuals and populations develop over time. A challenging environment can also shape the genomic landscape that underlies a population’s adaption to weather, resources, and predators [1–3]. These variables can take many abiotic and biotic forms, all with varying levels of intensity leading to a complex balance of genetics and environment. Climate change, especially in the form of weather extremes, has the ability to disrupt this balance and place a population under increased environmental stress [4–7]. For livestock production, this shift in climate driven environmental stressors has been detrimental to commercial traits [8–10]. Climate change has led to higher temperatures and drought, contributing to losses in livestock production worldwide related to reduced reproduction, growth, and immune function [8–10]. For example, high ambient temperatures can operate as a primary environmental stressor. Environmentally stressed chickens can experience oxidative-stress, lipid peroxidation, disruption of internal energy balance, and immunosuppression [10–17]. A major cellular effect caused by multiple environmental stressors is the generation of reactive oxygen (ROS) species that leads to oxidative stress and lipid peroxidation. This is brought about by changes in intracellular oxidation, which results in a state of imbalance between ROS and antioxidants [12–15]. Oxidative stress can be detrimental to gene expression causing post-transcriptional changes to signaling genes [18, 19] disrupting the health of an animal at the genetic level. Oxidative stress in chickens can also cause endothelial dysfunction and vasoconstriction [20–22]. However, the differences in how chickens respond to stressors may depend upon their evolutionary course and how it was influenced by selective pressure to adapt for survival. Constant selection on these survival traits can lead to the presence of genomic signatures that indicate what genomic regions responded to selective pressure. Selection for survival traits can lead to reduction in the variability around genomic regions associated with that trait. This reduction in variability, referred to as a selection signature or selective sweep, can be detected and examined for its biological importance. Researchers have employed multiple methods for detecting the presence of these areas under selection by measuring the reductions in diversity of a genomic region [23, 24] both within and between populations. Studies examining selection signatures within populations have used methods based on the reduction of heterozygosity to detect selection and identify important domestication loci [25, 26] for traits related to reproduction, growth, feeding behavior, and skin color. Other studies have successfully used F_st_ measurements, pooled heterozygosity, and extended homozygosity to investigate the chicken genome for signatures of selection underlying key economic traits such as growth and egg production in commercial breeds [27–29]. Through comparisons of both wild and domesticated chickens these studies have been able to separate traits driven by natural and artificial selection. Studies have also been conducted on indigenous chicken breeds that have uncovered evidence of possible independent selection events towards pathways related to niche survival environments, such as low oxygen at high altitudes [30] that would normally be considered as stressful.

The current study examined indigenous chicken ecotypes from the countries of Uganda and Rwanda for the presence of genomic signatures that may indicate that selective pressure from their environment helped shape the genetics underlying their adaption to various stressors. The two countries have environments that present many challenges, such as weather and food availability and quality, which may have led to chickens adapted to pressure from their environment. In addition to the indigenous birds, we also examined Kuroiler chickens in Uganda. Kuroilers were bred in India to be tolerant of heat, dual-purpose for meat and egg production, and with the ability to scavenge when food is scarce [31]. Studying populations that have developed under natural and artificial selection for a challenging environment may reveal genomic signatures related to these populations’ mechanisms of tolerance, resistance, or resilience. This may lead to a greater understanding of the genomic control of response to environmental stressors and aid in breeding of animals that are better able to tolerate stressors related to harsh environments and shifting climate patterns.

## Methods

### Sample collection

Blood samples were collected from 196 African chickens: Ugandan (*n* = 72), Rwandan (*n* = 100), and Kuroilers (*n* = 24). Kuroilers, originally imported from India, were sampled from farms in Uganda. Five physically distinct farms were sampled in each sampling area to make up a single geographically defined ecotype, in an attempt to reduce stratification. There were six ecotypes (30 farms) for Rwanda from the areas of Huye, Kicukiro, Kirehe, Musanze, Nyagatare, and Rubavu. For Uganda there were three ecotypes (15 farms) from the areas of Kamuli, Luweero, and Masaka. Blood was collected using FTA cards (Additional file 1: Table S1).

### Genotyping and quality control

Genotyping was conducted at GeneSeek (Lincoln, NE) using the Affymetrix Axiom ® 600 k Chicken Genotyping Array. SNPs were put through a quality control step in Plink [32, 33] based on the parameters of > 97 % call rate (-geno 0.03) and minor allele frequency (MAF) > 0.02. After filtering, 506,965 total SNPs remained. A total of 476,106 autosomal SNPs were available for downstream analyses.

### Population stratification analysis

Hierarchical clustering analysis of the genotype data in JMP ® (www.jmp.com) was used to determine the relationships between the samples. This cluster analysis was run using the ward method [34]. PLINK [32, 33] was used to construct a multi-dimension scaling plot (MDS-plot) to examine population structure for stratification. MDS plots for this analysis were based on 196 × 196 matrix of genome-wide Identity-By-State (IBS) scores calculated based on pairwise comparisons of the genetic distances for all individuals. The APE [35] and PhanGorn [36] R packages were used to examine the relatedness of the birds by phylogenetic analysis using neighbor-joining (NJ) distance based tree construction methods (data not shown). Visualization of the corresponding trees was done using FigTree (http://tree.bio.ed.ac.uk/software/figtree) and Ninja (http://mesquiteproject.wikispaces.com/Additional+Mesquite+Packages). IBS values and inbreeding coefficients were calculated within Plink. Principal component analysis (PCA) was done using SNP and Variant Suite (SVS) [37] and JMP ® (www.jmp.com). Shared ancestry was also explored using the Admixture software [38] set at varying values of k, ranging from 1 to 9 to represent the number of ecotypes sampled, with a final optimal *k* = 3.

### Runs of homozygosity analysis

Runs of homozygosity (ROH) analyses were carried out in Plink [32, 33] to examine genomic regions that harbor alleles driven to fixation using a SNP based sliding window approach. Runs of homozygosity were calculated for each individual and any ROH that overlapped between individuals within and between populations. For the individual and overlapping ROH analysis, a run was defined in Plink as  ≥ 250 SNPs, density of 50 kb/SNP, allowed gap of 1000 kb, and 3 heterozygous calls allowed within a run. The analysis focused on the overlapping ROH that were also analyzed using the additional parameters of allelic match threshold of 0.95 identity and 20 or more informative SNPs. The overlapping ROH are regions that overlapped across all populations and contained 10 or more individuals. Consensus regions between populations were defined as the region that was common to every bird, irrespective of length of the ROH, that Plink assigned to a “pool” because of shared overlapping ROH. The analysis of the overlapping/consensus regions was based on all samples as a whole and a “pool” of individuals sharing overlapping/consensus ROH was made up of birds from all three populations. A gene ontology enrichment analysis was conducted on a gene list from the ROH consensus regions [39, 40]. The gene list is based only on genes mapped to within the consensus region of the ROH. Gene ontology enrichment analysis results were considered statistically significant at a FDR cut-off *< 0.15*.

### Analysis of putative selection signatures

Sample haplotypes were phased using FastPhase [41] for downstream analysis of selection signatures. The R package REHH [42] was used to calculate integrated haplotype score (iHS) and standardized log-ratio of the integrated EHHS (iES) between pairs of populations (Rsb) values to examine the populations for SNPs that displayed signals of selection. Both iHS and Rsb values were log transformed to normalize the data and calculated as per the method established by Voight et al., 2006 [43]. Statistical significance of iHS values were determined by use of the-log *p*-values generated by the REHH software package. The -log *p*-values were not adjusted for multiple test correction because all multiple test correction procedures proved to be too conservative due to the number of tests exceeding 400,000. To address the issue of statistical significance a very stringent nominal *p*-value (*α = 0.001, −log α = 4*) was set and iHS data was then ranked from lowest to highest. After ranking, the *p*-value (*α = 0.001, −log α = 4*) was used as the cut-off for all samples and populations. iHS values were considered extreme at *iHS > |3.29|* because this was the lowest iHS value at the *p*-value cut-off. Pairwise comparisons between populations were examined using the Rsb statistic. *P*-values for the Rsb statistic were low enough to be subjected to multiple test correction using a FDR cut-off of *< 0.05*. The use of a lower FDR was also necessary to reduce the number of significant results for a more focused downstream analysis. To carry out gene ontology term enrichment, SNPs were annotated to genes of interest using the Affymetrix NetAffx™ Analysis Center which, when supplied with a list of markers from one genotyping platform, will give a list of genes within, upstream, or downstream of a gene in that region.

### Gene ontology enrichment analysis

Gene ontology (GO) term enrichment was performed using (GO)TermFinder [39]. Visualization and reduction of redundant terms of the GO enrichment results were carried out using ReVigo [40]. The significant GO terms were filtered for redundant terms to produce a focused list of the functions and processes under selection. The FDR for the enrichment tests was set at *< 0.15.* This cut-off was set higher than the threshold for the iHS or Rsb tests since it was a separate independent test. Another point that prompted the use of a higher FDR was the lack of annotations for the chicken genome, which effectively reduced the overall number of genes that could be analyzed.

## Results

### Population structure analysis

**Fig. 1:**
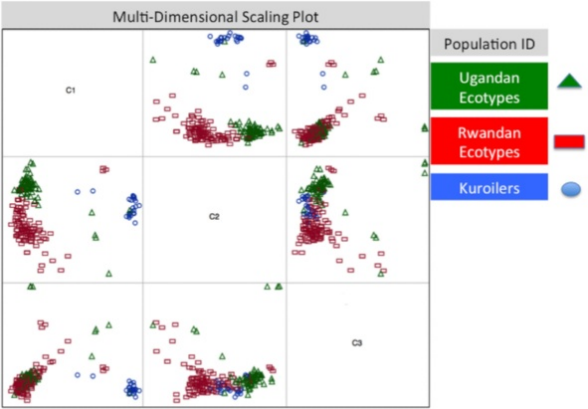
Multi-dimensional scaling plot showing distinct sampling populations. The multi-dimensional scaling plot was constructed using genomic distances to examine for the presence of population stratification

**Fig. 2:**
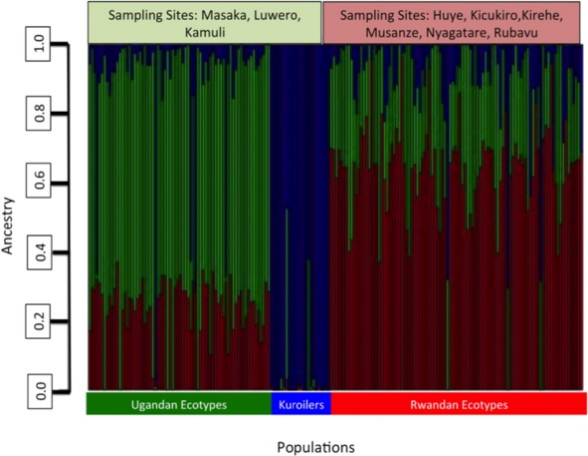
Admixture plot showing mixed ancestry between populations and individuals. *Green* denotes Ugandan ecotypes, *red* denotes Rwandan ecotypes, *blue* denotes Kuroilers. Based on optimal *k* = 3

The MDS plot (Fig. 1) showed overlap among Ugandan, Rwandan, and Kuroiler populations. The Ugandan and Rwandan ecotypes showed the highest degree of overlap between populations, with the Kuroilers showing the most discrete clustering of individuals. The amount of admixture, based upon identity by state, within each country (3 Ugandan ecotypes, 6 Rwandan ecotypes) showed crossover between sampling areas (Fig. 2). The clustering analysis based on the SNP genotyping calls indicated that the ecotypes (sampling location) assigned to the birds showed shared ancestry of genotypes between individuals across populations and ecotypes. These results were visualized as an admixture plot that also showed evidence of shared ancestry (Fig. 2).

### Examination of runs of homozygosity (ROH)

The number and extent of individual ROH differed widely among the populations (unpublished data). Kuroilers had the fewest chromosomes that contained ROH, while the combined Ugandan ecotypes were the only population to have ROH on every chromosome except chromosome 16. Chromosome 16 showed no evidence of ROH based on the parameters in any of the populations. The median length of the ROH in a population was longest in Ugandan and shortest in Kuroiler. The amount of the genome covered by ROH per individual ranged from ~2 % to 40 % (unpublished data).

### Examination of ROH overlapping consensus regions

The data were also analyzed for consensus overlapping ROH regions between all three populations that were identical by coordinates (Table 1). For the consensus ROH, the macro-chromosomes had the highest number of overlapping regions, span (kb), and number of SNPs comprising the runs on a chromosome. A gene list was created for the combined consensus regions and then analyzed using GO enrichment to determine over-enriched GO terms. There were 150 consensus overlapping ROH meeting criteria that harbored 343 genes that were used to conduct the GO enrichment analysis. The consensus ROH varied widely in the number of genes they contained, ranging from 0 to 24 genes within a given ROH. Statistically significant (*FDR < 0.15*) GO terms for biological processes and molecular functions related to both endogenous and external stressors (Table 2). Some of the most striking over-enriched gene ontology processes included regulation of cellular response to stress (GO:0080135), regulation of reactive oxygen species metabolic process (GO:2000377), regulation of apoptotic process (GO:0042981), and calcium ion transmembrane transport (GO:0070588) (*FDR < 0.15*). Over-enriched molecular functions that may be related to oxidative stress induced by the environment included calcium ion binding (GO:0005509), protein serine/threonine kinase activity (GO:0004674), and transforming growth factor beta receptor binding (GO:0005160) (Table 2).

**Table 1:** Summary of ROH consensus regions present amongst all 3 populations, by chromosome

Chr	N (Consensus)	Mean (# of birds)	Mean ROH length (Kb)	Sum ROH length (Kb)	Mean number Of SNPs	Sum Of SNPs
1	46	13.83	298.88	13748.66	124.37	5721
2	20	16.70	572.33	11446.66	198.65	3973
3	17	12.65	326.66	5553.24	144.35	2454
4	21	17.19	153.77	3229.19	64.00	1344
5	9	14.33	482.82	4345.38	153.00	1377
6	4	10.75	256.77	1027.10	143.50	574
7	7	13.43	211.29	1479.02	102.14	715
8	6	21.33	218.13	1308.76	107.17	643
9	3	11.67	204.61	613.82	138.67	416
10	7	11.57	171.48	1200.37	158.29	1108
11	3	20.67	371.64	1114.93	207.00	621
12	1	12.00	1252.64	1252.64	778.00	778
13	1	10.00	984.15	984.15	408.00	408
14	1	18.00	1034.33	1034.33	645.00	645
15	2	10.50	239.67	479.33	182.50	365
19	2	13.00	511.86	1023.72	276.50	553

Each population is represented in each consensus group by at least one bird. Only consensus groups with 15 or more individuals shown. Consensus regions in between flanking markers were annotated for genes and coding non-synonymous and splice site category SNPs

**Table 2:** Gene ontology (GO) enrichment of consensus ROH analysis^a^

GO: ID	Go: term
GO:0006915	Apoptotic process
GO:0002209	Behavioral defense response
GO:0001662	Behavioral fear response
GO:0070588	Calcium ion transmembrane transport
GO:0071345	Cellular response to cytokine stimulus
GO:0071495	Cellular response to endogenous stimulus
GO:0071396	Cellular response to lipid
GO:0033554	Cellular response to stress
GO:0019221	Cytokine-mediated signaling pathway
GO:0006281	DNA repair
GO:0007631	Feeding behavior
GO:0007599	Hemostasis
GO:0031663	Lipopolysaccharide-mediated signaling pathway
GO:0032873	Negative regulation of stress-activated MAPK cascade
GO:0070303	Negative regulation of stress-activated protein kinase signaling cascade
GO:0016310	Phosphorylation
GO:0042981	Regulation of apoptotic process
GO:0080135	Regulation of cellular response to stress
GO:0001959	Regulation of cytokine-mediated signaling pathway
GO:0043408	Regulation of MAPK cascade
GO:2000377	Regulation of reactive oxygen species metabolic process
GO:0080134	Regulation of response to stress
GO:0032319	Regulation of rho gtpase activity
GO:1901700	Response to oxygen-containing compound
GO:0009314	Response to radiation
GO:0006950	Response to stress
GO:0023014	Signal transduction by phosphorylation
GO:0033209	Tumor necrosis factor-mediated signaling pathway
GO:0042060	Wound healing
GO:0003684	Damaged DNA binding
GO:0005246	Calcium channel regulator activity
GO:0005488	Binding
GO:0008289	Lipid binding
GO:0030234	Enzyme regulator activity
GO:0005509	Calcium ion binding
GO:1901363	Heterocyclic compound binding
GO:0005160	Transforming growth factor beta receptor binding
GO:0005543	Phospholipid binding
GO:0004674	Protein serine/threonine kinase activity
GO:0035258	Steroid hormone receptor binding
GO:0008083	Growth factor activity

Subset of GO terms that could putatively affect responses to environmental stressors. ^a^All terms were statistically significant at FDR *< 0.15*. This analysis lends evidence to support that all three populations experienced selective pressures for variants effecting stress response, immune response, and behavior

### Genes under putative selection within populations

**Fig. 3:**
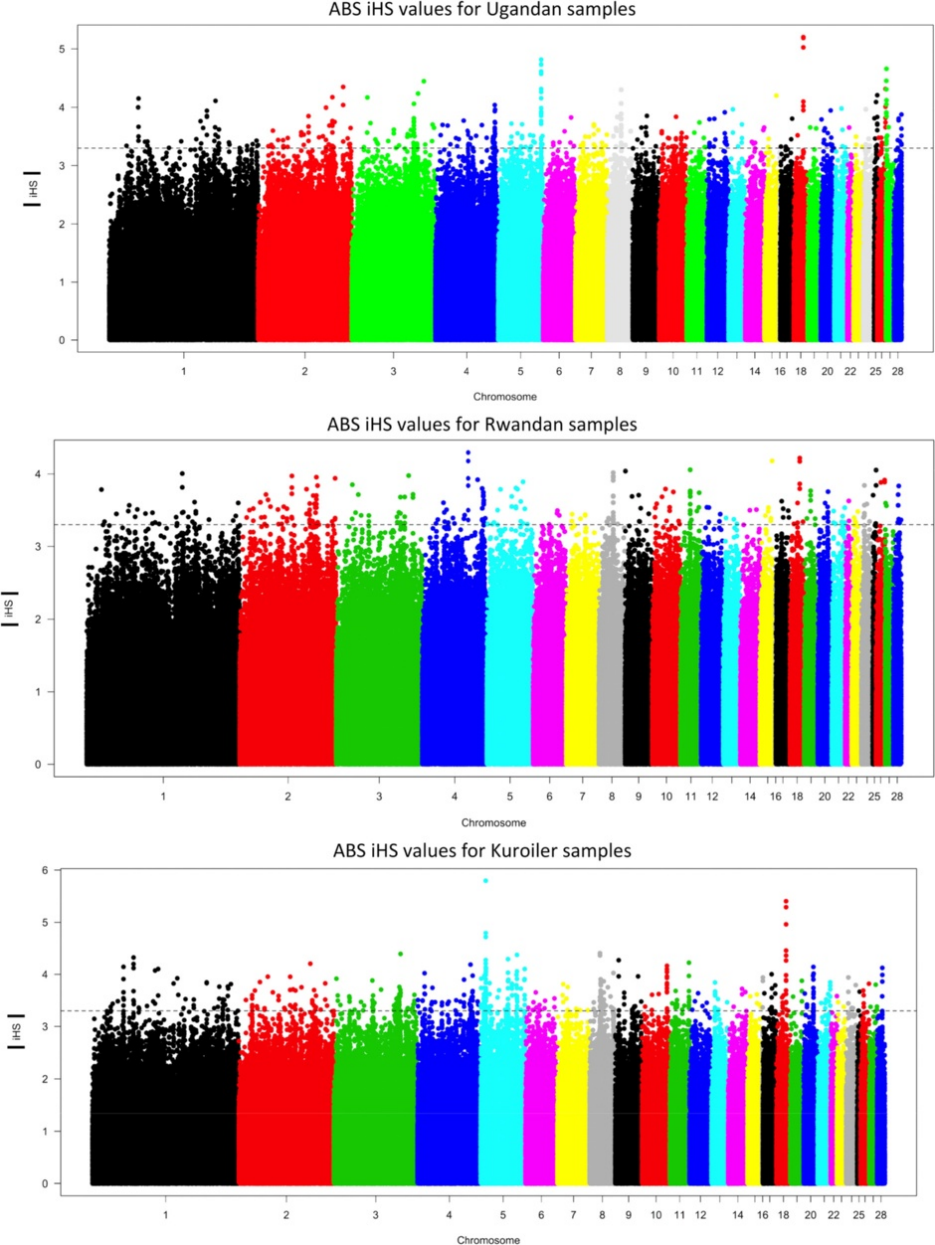
Manhattan plots of iHS values for each population. The *horizontal lines* represent the *p*-value cut-off for significance (< *0.001*). iHS scores were recorded as absolute values. iHS cut-off for significance *> |4|* for variants to be considered as signal of selection

**Fig. 4:**
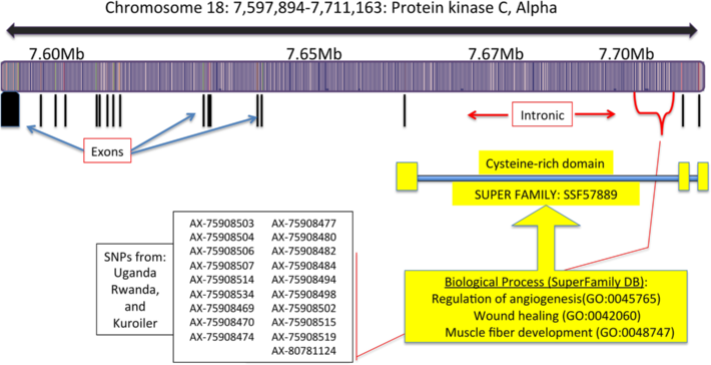
Statistically significant SNPs annotated to *PRKCA* fall within an intronic region of the gene near a cysteine-rich domain superfamily [86]. Variants are located near the protein kinase cysteine-rich domain, a group of small proteins that have functions possibly involved in the response to environmental stressors

The strongest iHS signal (*p< 0.001; iHS > |4|*) across all three populations was on chromosome 18 in variants annotated to intronic regions of the protein kinase C alpha (*PRKCA*) gene (Figs. 3 and 4) (Additional file 2: Table S2). It was the strongest overall signal in the Ugandan birds, and second strongest signal in the Rwandan and Kuroilers (*p< 0.001; iHS > |4|*). There were also strong selection signals that were unique to each of the three populations. In the Uganda ecotypes the gene cyclin-dependent kinase inhibitor 3 (*CDKN3*) on chromosome 5 had the next highest iHS value (*iHS = |4.82|*) with 41 statistically significant variants (*p < 0.001*). The gene deleted in liver cancer 1 *(DLC1)* on chromosome 4 had the highest iHS value in the Rwandan birds (*iHS = |4.29|*) supported by 44 statistically significant variants (*p < 0.001*). In Kuroilers, variants annotated to adrenomedullin (*ADM*) (iHS = |5.79|) on chromosome 5 and glutamate-cysteine ligase, modifier subunit (*GCLM*) on chromosome 8 (iHS = |4.40|) had the highest iHS values (Additional file 3: Table S3).

### Putative genes under selection between populations

The populations were also compared pairwise by means of the Rsb statistic to identify additional genomic regions under selection. Statistical significance (*FDR < 0.05*) and the highest Rsb value (*>|7|*) were used to determine genes under possible selection. The Ugandan ecotypes vs. the Rwandan ecotypes showed selective pressure on chromosome 13 for Gamma-aminobutyric acid receptor, gamma-2 *(GABRG2*). Also annotated to the highest Rsb value on chromosome 13 was the gene teneurin-2 isoform 1 (*TENM2).* On chromosome 11, Beta, beta-carotene 15,15'-monooxygenase *(BCMO1)* reached statistical significance along with having a Rsb above |7|. The Ugandan and the Rwandan ecotypes both yielded similar results when compared to the Kuroilers. The strongest selection occurred on chromosome 1 with the gene olfactomedin 4 (*OLFM4).*

### Gene set enrichment analysis

#### ROH

Gene ontology (GO) enrichment was conducted using a list composed of genes located between the flanking markers of the ROH consensus regions (Table 2). The GO enrichment was subjected to a FDR *< 0.15* then filtered for redundancy of terms using the software Revigo [40], which produced a list of significant GO terms related to selective pressures for variants effecting oxidative and cellular stress, immune response, and behavior. The GO enrichment also provided information on possible molecular functions and biological processes under selection related to calcium, lipid, and kinase activity and binding. Significance was also reached for processes related to UV radiation and DNA repair, possibly as a result of the birds living at the equator.

#### iHS

A GO enrichment was conducted using a list of genes annotated to statistically significant (*p < 0.001*) variants on the chip. Statistically significant (*FDR ≤ 0.15*) enriched GO terms relating to a host of environmental stressors emerged from the analysis. Regions of the genome in each population showed evidence of selective pressure on biological processes related to multiple stress-related signaling pathways. There was also evidence of selective pressure on molecular functions involved in kinase and lipid activity. In the Ugandan ecotypes and Kuroilers GO enrichment showed selective pressure on protein kinase activity (Additional file 4: Figure S4). The Rwandan and Kuroiler populations were both associated with *PRKCA* through the GO term protein kinase, this was not the case in the Ugandan ecotypes (Tables 3, 4 and 5).

**Table 3:** Gene ontology (GO) enrichment of statistically significant (*FDR < 0.15*) GO terms for the Ugandan ecotypes

Population	GO ID	Gene ontology term	Genes under GO term	Related environmental stressor(s)	Reference
Ugandan Ecotypes	GO:0065008	Regulation of biological quality	PDIA5, CADPS2, TNIP2, CNOT6L, DISP3, ABCA4, PRKCA, SIN3A, PEX11A, DLG1, MC4R, RC3H1, NPY, SMAD1, CNGA2, RCOR1, RAP1GDS1, NAT8L, BICD2, SYNE1, ATP9A, STX12	Growth response to Environmental Stress	UniProt [67], Jenuth 2000 [69, 77]
	GO:0001816	Cytokine production	DLG1, TRIB2, MAP3K7, EIF2AK2, SNAI2	Pathogen/Predator	Welc et al. 2013 [56]
	GO:0043122	Regulation of I- kappaB kinase/NF-kappaB signaling	TNIP2, ZFAND6, ZDHHC17, MAP3K7	Inflammation/Oxidative Stress	Evans et al. 2002 [46], Perkins et al. 2007 [55] Salminen et al. 2008 [87], Tam et al. 2012 [88]
	GO:0016627	Oxidoreductase activity, acting on the CH-CH group of donors	TECRL, DYPD	Oxidative stress	UniProt [67], Jenuth 2000 [69, 77], Lee et al. 2007 [89]
	GO:0005215	Phospholipid transporter activity	ABCA4, ATP9A	Lipid Metabolism, phospholipid transfer to membrane	UniProt [67], Jenuth 2000 [69, 77]
	GO:0080134	Regulation of response to stress	TNIP2, PSMB4, MAP3K7, SIN3A, MID1, EIF2AK2, SNAI2	Oxidative/Metabolic/Environmental Stress	UniProt [67], Puvadolpirod et al. 2000 [48], Li et al. 2011 [70], Yen et al. 2014 [90]
	GO:1901224	Positive regulation of NIK/NF-kappaB signaling	RC3H1, MAP3K7, EIF2AK2	Inflammation/Oxidative Stress	Evans et al. 2002 [46], Perkins et al. 2007 [55], Salminen et al. 2008 [87], Tam et al. 2012 [88]
	GO:0031098	Stress-activated protein kinase signaling cascade	MAP3K7, MID1, EIF2AK2	Oxidative/Environmental Stress	Li et al. 2011 [70], Yen et al. 2014 [90]
	GO:0006915	Apoptotic process	TNIP2, SUDS3, ROBO1, ZFAND6, DNAJC5, MAP3K7, LCMT1, SIN3A, GRK5, EIF2AK2, SHQ1, CHL1, COMP, LFABP, SNAI2	Oxidative/Environmental Stress	Galvez et al. 2001 [47]
	GO:0032874	Positive regulation of stress-activated MAPK cascade	MAP3K7, MID1, EIF2AK2	Oxidative/Environmental Stress	UniProt [67], Jenuth 2000 [69, 77]
	GO:0006950	Response to stress	PDIA5, CADPS2, TNIP2, PSMB4, MAP3K7, UBE2T, SIN3A, EIF2AK2, MMP7, CXCR4, MID1, PAX-7, LFABP, SMAD1, SNAI2	Oxidative/Environmental Stress	UniProt [67], Puvadolpirod et al. 2000 [48], Li et al. 2011 [70], Yen et al. 2014 [90]
	GO:0009719	Response to endogenous stimulus	MC4R, GRB10, DISP3, MAP3K7, SMAD1, STMN2, SNAI2	Oxidative/Environmental Stress	Evans et al. 2002 [46]
	GO:0034976	Response to endoplasmic reticulum stress	PDIA5, EIF2AK2	Oxidative/Environmental Stress	Evans et al. 2002 [46], Zhang et al. 2008 [54]

**Table 4:** Gene ontology (GO) enrichment of statistically significant (*FDR < 0.15*) GO terms for the Rwandan ecotypes

Population	GO ID	Gene ontology term	Genes under GO term	Related environmental stressor(s)	Reference
Rwandan Ecotypes	GO:0004697	Protein kinase C activity	PRKCA, PRKD1	Oxidative stress	UniProt [67], Jenuth 2000 [69, 77]
	GO:0046649	Lymphocyte activation	DLG1, GPAM, ZBTB16, PREX1, CD151	Pathogen/Predator	Flint et al. 2011 [91]
	GO:0006954	Inflammatory response	PSMB4, UACA	Pathogen/Predator	Evans et al. 2002 [46], Zhang et al. 2008 [54]
	GO:0006950	Response to stress	SEL1L, PSMB4, SIRT6, NEK6, BCAS3, GPAM, LIG3, UACA, PRKD1, SNAI2, SMAD1	Growth response to Environmental Stress	UniProt [67], Puvadolpirod et al. 2000 [48], Li et al. 2011 [70], Yen et al. 2014 [90]
	GO:0007249	I-kappaB kinase/NF-kappaB signaling	UACA, ZDHHC17, NEK6, PRKD1	Inflammation/Oxidative Stress	Evans et al. 2002 [46], Perkins et al. 2007 [55], Salminen et al. 2008 [87], Tam et al. 2012 [88]
	GO:0034599	Cellular response to oxidative stress	UACA, PRKD1	Oxidative/Environmental Stress	UniProt [67], Jenuth 2000 [69, 77]
	GO:0009719	Response to endogenous stimulus	HPGD, GRB10, CITED3, GABRB3, BCAS3, SMAD1, STMN2, SNAI2	Oxidative/Metabolic stress	Evans et al. 2002 [46]
	GO:0048010	Vascular endothelial growth factor receptor signaling pathway	GRB10, PRKD1	Oxidative/Metabolic Stress	Koch et al. 2012 [57]
	GO:0043281	Regulation of cysteine-type endopeptidase activity involved in apoptotic process	DLC1, RFFL, UACA	Metabolic stress	Galvez et al. 2001 [47]
	GO:0065008	Regulation of biological quality	DLC1, PRKCA, RAP1GDS1, DLG1, GPAM, FRMPD4, GRM8, CRTC1, NPY, PTPN3, STX12, SMAD1	Growth response to Environmental Stress	UniProt [67], Jenuth 2000 [69, 77]
	GO:0006915	Apoptotic process	ZBTB16, DLC1, RFFL, SUDS3, DNAJC5, SHQ1, GPAM, UACA, GABRB3, UTP11L, SNAI2	Metabolic stress	Galvez et al. 2001 [47]

**Table 5:** Gene ontology (GO) enrichment of statistically significant (*FDR < 0.15*) GO terms for the Kuroilers

Population	GO ID	Gene ontology term	Genes under GO term	Related environmental stressor(s)	Reference
Kuroilers	GO:0060584	Regulation of prostaglandin-endoperoxide synthase activity	EDN2, EDN1	Oxidative stress/Pathogen	Lee et al. 2007 [89]
	GO:0007631	Feeding behavior	HCRTR2, HTR1B, GRIN1, NPY1R, ASIP	Nutrient stress	UniProt [67], Jenuth 2000 [69, 77]
	GO:0032693	Negative regulation of interleukin-10 production	TRIB2, IL-12B	Pathogen	UniProt [67], Jenuth 2000 [69, 77]
	GO:0051341	Regulation of oxidoreductase activity	ACVR2A, EDN2, EDN1, SNCA	Oxidative stress/Pathogen	UniProt [67], Jenuth 2000 [69, 77], Lee et al. 2007 [89]
	GO:0007618	Mating	ACVR2A, SERPINE2, GRIN1	Survival Behavior	UniProt [67], Jenuth 2000 [69, 77]
	GO:0016627	Oxidoreductase activity, acting on the CH-CH group of donors	IVD, CPOX, ACAD9, DHCR24	Oxidative/Environmental Stress	UniProt [67], Jenuth 2000 [69, 77], Lee et al. 2007 [89]
	GO:0006950	Response to stress	ENTPD1, ETFDH, WNT5A CHID1, RBBP5, NEDD4, NSMCE2, GLP1R, STX2, LEAP2, RAB23, DEAF1, TNFSF11, PPP2R5C, DRD4, ZC3HAV1, CDH8, PPAP2B, GTF2H5, MCPH1, EDN1, FAM175B, GCLM, JAK1, UBXN2A, MID1, BATF, CPEB2, IL12B, PIK3AP1, SOX2, SNCA, CITED2, CHAC1, DTL, HIPK3, NOS1, STT3B, PDIA4, SBNO2, NUAK1, CDC7, SERPINE2, ULK1, MASP2, WFS1, DHCR24, PRKD1	Oxidative/Metabolic/Environmental Stress	UniProt [67], Puvadolpirod et al. 2000 [48], Li et al. 2011 [70], Yen et al. 2014 [90]
	GO:0080134	Regulation of response to stress	WNT5A, CHID1, PIK3AP1, IL12B, STX2, SNCA, HIPK3, TNFSF11, ZC3HAV1, PPAP2B, SBNO2, NUAK1, MCPH1, EDN1, SERPINE2, ULK1, WFS1, MID1	Oxidative/Metabolic/Environmental Stress	UniProt [67], Puvadolpirod et al. 2000 [48], Li et al., 2011, Yen et al., 2014
	GO:0033554	Cellular response to stress	WNT5A, RBBP5, BATF, CPEB2, NEDD4, NSMCE2, SNCA, CHAC1, CITED2, DTL, HIPK3, RAB23, TNFSF11, PPP2R5C, PDIA4, GTF2H5, NUAK1, FAM175B, EDN1, CDC7, ULK1, WFS1, UBXN2A, MID1, PRKD1	Oxidative/Metabolic/Environmental Stress	UniProt [67], Puvadolpirod et al. 2000 [48]
	GO:0045859	Regulation of protein kinase activity	TNFSF11, DRD4, WNT5A, TGFBR2, RAPGEF2, PDGF, PIK3CA, KIAA1199, LATS2, IL12B, EDN1, PAQR3, SNCA, GTPBP4, HIPK3	Oxidative/Metabolic/Environmental Stress	UniProt [67], Jenuth 2000 [69, 77]
	GO:0003100	Regulation of systemic arterial blood pressure by endothelin	EDN2, EDN1	Oxidative/Metabolic Stress	Endemann et al. 2004 [20], Gomez et al. 2007 [21]
	GO:0048010	Vascular endothelial growth factor receptor signaling pathway	FIGF, NEDD4, GRB10, PRKD1	Oxidative/Metabolic Stress	Koch et al., 2012 [57]
	GO:0004674	Protein serine/threonine kinase activity	CDC42BPB, DCLK1, TGFBR3, TGFBR2, PRKCA, LATS2, ACVR2A, NUAK1, CDC7, ULK1, CDC2L1, HIPK3, VRK1, PRKD1	Oxidative/Metabolic/Environmental Stress	UniProt [67], Evans et al. 2002 [46], Perkins et al. 2007 [55], Salminen et al. 2008 [87], Tam et al., 2012 [88]
	GO:0009628	Response to abiotic stimulus	GPR65, GRIN1, CASP8, IL12B, CPEB2, SOX2, NEDD4, TNFRSF8, CITED2, DTL, ER81, NOS1, DEAF1, CNGA2, CDH8, MME, GTF2H5, HR1B, SERPINE2, EDN1, FAM175B	Oxidative/Metabolic/Environmental Stress	Mashaly et al. 2004 [92], Mujahid et al. 2005 [16]
	GO:0004672	Protein kinase activity	CDC42BPB, DCLK1, PRKCA, HIPK3, VRK1, ROR1, TRIB2, TGFBR3, TGFBR2, LATS2, ACVR2A, NUAK1, CDC7, ULK1, CDC2L1, PEAK1, JAK1, RPS6KC1, PRKD1	Oxidative/Metabolic/Environmental Stress	UniProt [67], Jenuth 2000 [69, 77]

## Discussion

### Population structure and admixture of populations indicate mixed genetic backgrounds

The observed population structure could partially be a result of using IBS values, which are based on the likelihood that identical alleles inherited by two individuals came from the same parent. This can lead to some uncertainty due to not having pedigree information, but it still allowed us to capture information on the population structure [44]. The amount of overlap seen across ecotype and populations may stem from flock management that allows unrestricted inter-mating of chickens from different genetic backgrounds. The admixture may be due to individuals having ancestors in multiple source groups that contribute to the shared ancestry. Another contributor to this admixture may be related to the movement of trade and selection parameters used by farmers to purchase new birds at market.

### Possible factors contributing to signatures of selection

There are multiple environmental challenges that could lead to the occurrence of the discovered selection signals in the three populations. The geographic location of Uganda and Rwanda is near the equator and in tropical climates (Additional file 5: Figure S5). This, coupled with smallholder farm practices, places the studied birds in situations in which they would likely contact multiple environmental stressors that may affect their fitness. This would include challenges to their immune system from pathogens in the absence of vaccination. Also the equatorial locations of the samplings were exposed to high ultraviolet rays. The birds may also have experienced nutritive stress brought on by sub-optimal food in an environment in which birds must forage for food. The birds used in this study would have adapted to the environment over the years, likely through changes in allele frequency of a beneficial or detrimental allele.

### Evidence of common signatures of selection across ecotypes for stress response

In livestock species, as well as humans and mice, environmental stressors such as pathogens or non-optimal temperatures can create cellular oxidative stress by generating reactive oxygen and nitrogen species effecting calcium signaling, apoptosis, vascular plasticity, growth and immune functions [16, 22, 45–50]. The chickens in our study displayed evidence of population similarities in how selection may have affected their response to environmental stress. Signatures are related to genes and signaling pathways involved in the reduction of ROS through utilization of Ca^2+^, lipids, and phosphorylated kinases. If the mobilization of Ca^2+^ is part of a prolonged response to chronic stressors it is possible that birds that scavenge for food have to make trade-offs in Ca^2+^ usage. An eggshell can contain a concentration 1.99 M Ca^2+^ [51]. Signal transduction of Ca^2+^ stored in the endoplasmic reticulum can be identified by increased cytoplasmic Ca^2+^ concentrations of 300–500nM [52]. The Uganda population showed GO terms related to endoplasmic reticulum (ER) stress response where Ca^2+^ is stored. Despite being such a small amount (300–500 nM) this diversion of resources may hinder other functions dependent on calcium. This mobilization of calcium for stress tolerance could also reduce the ability of birds to produce structurally competent shells and limit egg production. Pathways affected by calcium were also upregulated in heat stressed broilers [53]. We also uncovered selective pressure on negative regulation of multiple kinases involved in stress responses that may function to reduce pro-inflammatory cytokines. This reduction of pro-inflammatory cytokines had a lowering effect on metabolism, reducing stress during an immune response [10, 46, 54–56]. The most marked effect of the selection was the enrichment of terms related to response to stress, especially those indicating selection for or against cellular, endoplasmic reticulum (ER), and oxidative stress [46, 54, 57]. Other processes that reached significance include cell-cell signaling (all populations), VEGF signaling, cytokine binding/production (Uganda and Rwanda), and inflammatory response (Rwanda) (Tables 3, 4 and 5). Selection on the genes and processes reported may be related to cyto-protective effects being used by chickens to tolerate heat as a general stress response, because it is a constant in their environment [58, 59].

### Selection pressure identified by ROH analysis indicate similar biological processes between populations

The results from the ROH analysis indicate that the populations have experienced selective pressure on their genomes from the environment. Runs of homozygosity (ROH) are defined as long stretches of homozygous genotypes within a genome, thought to be the result of consanguinity and identity by descent (IBD) inheritance [44, 60–62]. It is possible that ROH carry genes or variants under positive selection or that smaller ROH are related to the hitchhiking effects of selective sweeps [60, 63]. Analysis of ROH has been used to uncover alleles detrimental to health in humans and livestock [61, 63–65]. However, it may be possible that the ROH shared between the three populations point to conservation of alleles necessary for survival. These may be alleles that provide protection from oxidative and other stressors due to the environment. The existence of statistically significant hits for calcium ion activity and apoptotic regulation may, respectively, be related to kinase activation and amino acid recycling in the birds as a means of maintaining energy to put toward redox functions. It is possible that the birds are diverting resources to autophagy to conserve resources to put into increasing calcium activity to lower metabolic temperatures. This may be possible since it has been shown that blood Ca^2+^ levels are inversely related to body temperature [66].

### Genes putatively under selection from environmental challenges among and within ecotypes

Protein kinase C alpha (*PRKCA*) found on chromosome 18 appears as a strong signal in all three populations. The gene *PRKCA* is a calcium activated, phospholipid dependent kinase that is involved in gene expression, inflammation, prolactin secretion, as well as, the regulation of multiple cell processes including inflammation and wounding [67, 68] (Fig. 4). It is also upregulated in response to endothelial injury. In chicken, *PRKCA* carries out functions involved in gene expression and kinase activity, and is involved in prolactin secretion [67, 69]. The selective pressure on *PRKCA* may be related to the negative effect that a challenging environment has on overall body weight and feed efficiency [22, 70], which could indicate nutritive stress. In addition to its functions in livestock, *PRKCA* acts as a messenger to stimulate prolactin secretion [71], which in humans has been linked to psychosocial stress responses [72].

**Fig. 5:**
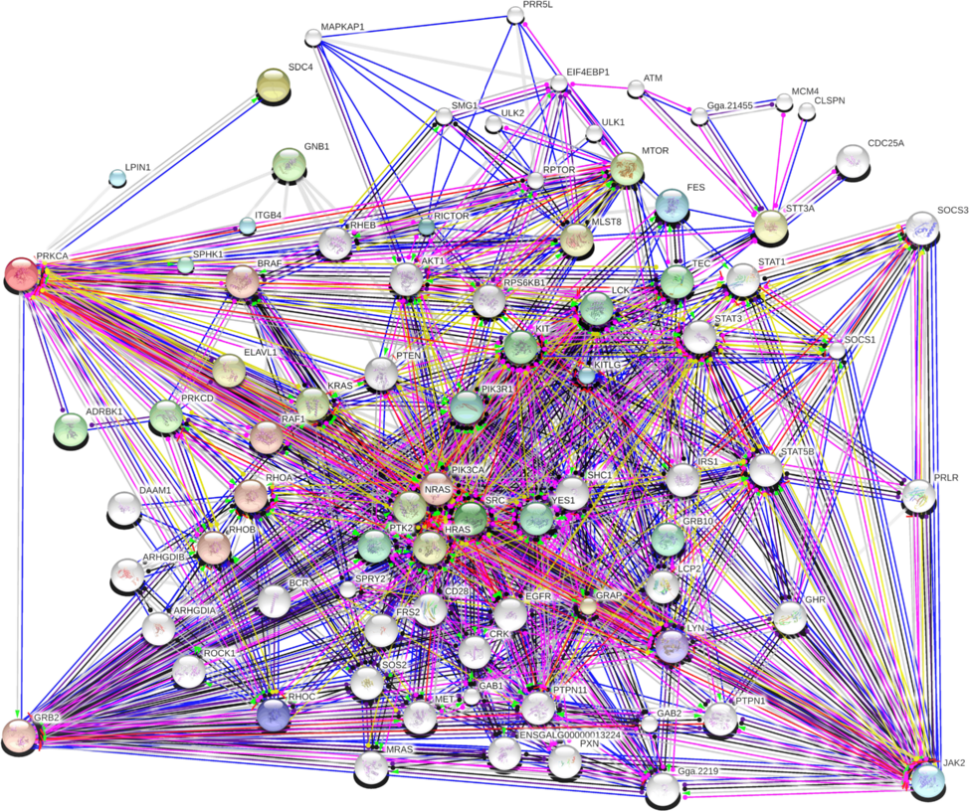
Network of predicted functional proteins associated with *PRKCA* created using STRING. Lines represent interactions that *PRKCA* has with predicted proteins in its network. Figure shows that *PRKCA* has protein-protein interactions with *SOCS1* and *SOCS3* that may be related to regulation of inflammation, while its interaction with *PRLR* may be related to stress pathways

Protein kinase C alpha is also involved in the biological process of prolactin secretion (GO:0070459) possibly through protein-protein interactions with the prolactin receptor (*PRLR).* This is interesting due to recent studies showing evidence of *PRLR’s* connection to heat tolerance in livestock [73, 74]. The interaction between *PRKCA* and *PRLR* indicates that both genes may contribute to the birds’ response to environmental stressors. In humans, *PRKCA* regulates induction of NF-kappa-B inhibitor alpha (*NFKBIA/IKBA*), which mediates host defense and inflammation responses, as well as plays a role in the activation of *MAPK* signaling and cyclin-dependent kinase *(CDK*) complex formation. Previous studies have indicated a role of *MAPK* and *NF-κB* pathways in the regulation of heat [70]. Regulation of *NF-κB* may be part of the response to inflammation triggered by ROS. The possible reduction in metabolic heat by *PRKCA* may be related the protein-protein interactions it has with suppressor of cytokine signaling 1 (*SOCS1*) and suppressor of cytokine signaling 3 (*SOCS3*) (Fig. 5). Both *SOCS1* and *SOCS3* help to battle ROS and inflammation and may play a role in the cascade needed to restore lost homeostasis [53, 75, 76]. Other *SOCS* genes have also been shown to respond under heat stress, which is a possible indication of a link between heat stress tolerance and cytokines [53].

The other kinase, cyclin-dependent kinase inhibitor 3 (*CDKN3*) found on chromosome 5 only appears as a strong signal in the Ugandan ecotypes. As a dual specific protein phosphatase, *CDKN3* functions to inhibit cell cycle processes by blocking kinases targeting cyclin-dependent kinase 2 (*CDK2)*, which causes cell cycle arrest and apoptosis in response to stress signals. The *CDK2* gene is also part of the FoxO signaling pathway, which regulates transcription factors involved in gene activation of physiological cellular processes such as apoptosis, glucose, oxidative and nutrient stimuli stress resistance. Another function of *CDKN3* is its ability to associate with cyclin C for activation and regulate different cell cycle events [67, 69, 77]. The selection pressure on *CDKN3* in the Ugandan ecotypes may be related to its part in cell cycle regulation and ability to interact with phosphotyrosine or phosphoserine residues [67]. It functions to inactivate *CDK2* which is part of a pathway engaged in DNA damage/telomere stress induced senescence, cell cycle arrest, and apoptosis in response to stress signals. To prevent senescence caused by oxidative stress, *CDKN3* may function to inhibit *CDK2*. Studies have shown that oxidative stress can trigger activation of Protein Tyrosine Kinases (*PTKs*), which leads to the release of intracellular calcium in in vitro chicken cells and rat macrophages [49, 50]. The gene *CDKN3* has also been shown to be considerably down regulated in mice undergoing oxidative stress from simulated environmental exposures [78].

In addition to the strong signal on chromosome 18, the Rwandan ecotypes also had a strong signal on chromosome 4 variants connected to the Rho GTPase activating protein, *DLC1.* This gene has functions involved in lipid binding and Rho GTPase activator activity and is also involved in negative regulation of stress fiber assembly. The gene *DLC1* is also involved in the activation of cysteine-type endopeptidase activity involved in the apoptotic process, as well as positive regulation of the execution phase of apoptosis [67, 69, 77, 79]. For the Rwandan ecotypes, *DLC1* may be involved in a population-specific mechanism of increasing cell migration and expanding cellular redox and lipid binding capabilities [67, 79, 80].

The gene with the strongest signal in the Kuroilers, *ADM*, is a hypotensive peptide that functions in hormone activity and in chicken is part of the biological processes of vasculogenesis (GO:0001570), positive regulation of angiogenesis (GO:0045766), and negative regulation of vasoconstriction (GO:0045906) [67, 69, 77, 81]. It also protects endothelial cells from cardiac stress [21]. In humans it is a vasodilator with functions that regulate fluid and electrolyte homeostasis and also has a role in the biological processes of response to cold (GO:0009409), hypoxia (GO:0001666), starvation (GO:0042594), LPS (GO:0032496), and wounding (GO:0009611) [45, 67, 82]. Other studies have shown *ADM* to also have anti-microbial, anti-apoptosis, and antioxidant functions [45, 67, 82, 83]. In the chicken, *ADM* has strong vascular modulation and functions as an antioxidant. Oxidative stress increases expression of inflammatory responses by endothelial cells [45, 68, 82] and *ADM* is shown to protect cardiovascular cells from oxidative damage [82, 83]. The selective pressure on this gene seen in the Kuroilers does not occur in the African ecotypes and may represent a result of artificial selection for stress tolerance in the Kuroilers during their development in India. The gene *GCLM* in chickens is part of the response to oxidative stress (GO:0006979), apoptotic mitochondrial changes (GO:0008637), and negative regulation of extrinsic apoptotic signaling pathway (GO:2001237). In humans, *GCLM* is also part of the response to nitrosative stress (GO:0051409) [67, 69, 77]. Nitrosative stress is caused by the formation of reactive nitrogen species (RNS) from cellular nitric oxide (NO) or it reacting with oxidative stress molecules to inflict cellular and vascular damage [84]. Other genes of interest that were in statistically significant regions of the genome based on the iHS values included *DnaJ* (*Hsp40*) homolog, subfamily C, member 5 (*DNAJC5*) and Collagen alpha-2(VI) chain (*COL6A2*). The heat shock protein *DNAJC5* appeared in all 3 populations and functions as a molecular chaperone and a negative regulator of neuron apoptosis. It is also part of the GABA synthesis, release, re-uptake and degradation pathway and the protein processing in endoplasmic reticulum pathway. Collagen alpha-2(VI) chain (*COL6A2*) only appeared in the Kuroiler samples and was the only stop-gain (high impact) variant to reach statistical significance based on the iHS analysis. It is involved in focal adhesion and is part of the collagen biosynthesis and modifying enzymes, collagen degradation, and integrin cell surface interaction pathways [67, 69].

### Pairwise comparisons of populations reinforced selection toward genes and functions related to oxidative stress

The comparison between the Ugandan and Rwandan ecotypes pointed selective pressure on gamma-aminobutyric acid A receptor, gamma 2 (*GABRG2),* an inhibitory neurotransmitter in vertebrates that mediates neuronal inhibition by binding to *GABA* receptors and opening an integral chloride channel. This is consistent with the results we observed in *DNAJC5*. Gamma-aminobutyric acid A receptor, gamma 2 (*GABRG2*) is also involved in the response to hypoxia in chickens [67, 69, 77]. The gene teneurin transmembrane protein 2 (*TENM2)* also plays a part in neural development and regulation of proper nervous system connections and carries out calcium-mediated signaling using intracellular calcium sources, hemophilic and heterophilic cell-cell adhesion [67, 69, 77]. The calcium functions of *TENM2* may help to stimulate the necessary calcium release to activate *PRKCA* into activation when stressors appear. On chromosome 11, beta, beta-carotene 15,15’-monooxygenase (*BCMO1),* functions as a mono-oxygenase activator involved in cellular redox reactions as scavengers of oxygen radicals for photo-protection [67, 69, 77]. *BCMO1* might experience selection related to protection from uv-induced DNA damage related to the generation of oxidative stress. When Ugandan and Rwandan ecotypes were compared to Kuroilers, the genes with the strongest signals for selection were the same but had different markers for the same gene regions in each comparison. Olfactomedin 4 (*OLFM4)* functions as a negative apoptotic factor and an extracellular matrix glycoprotein involved in cell adhesion. In chickens, it negatively regulates I-kappaB kinase/NF-kappaB signaling and the immune response. The between-population results for GO enrichment also reinforce the GO term results seen within population with slightly more emphasis on signaling, activation, and transport functions.

### Unique and shared features under selection in Kuroilers compared to native African ecotypes

Kuroilers showed a great deal of overlap with the African ecotypes in biological processes that reduce the effects of oxidative and metabolic stress. Unique to the Kuroilers were selection on prostaglandin-endoperoxide synthase activity, a target of *NF-κB* and negative regulation of interleukin-10 (*IL-10*) production. Both are a part of reduction of oxidative stress and have anti-inflammatory effects. The differences in the guided selection of the Kuroilers can be seen in the many growth and behavior related GO terms that are enriched within the Kuroiler population and not observed in the African ecotypes. In Kuroilers, the GO enrichment results from the iHS analysis have larger gene lists, which may signify that under artificial selective breeding for stress tolerance, larger genomic areas or QTL regions may have been selected upon. This may be the opposite of natural selection in the African ecotypes, which may be focused on smaller regions of the genome. This is reflected in the genes annotated to similar terms seen in the Ugandan and Rwandan populations related to stress, inflammatory responses, and apoptosis. The length of the natural selection experienced by the African populations may have affected this also. This would have led to more recombination events, leading to smaller genomic regions under selection than what was identified in the Kuroilers, which is a recently developed breed. It appears that all three populations have found ways through natural and artificial selection to tolerate environmental stressors. What this overlap in GO terms also shows is that both the artificial selection for stress tolerance (Kuroilers) and natural selection (Ugandan and Rwandan ecotypes) share a biological link. Previous studies showed evidence that exposure to one stressor, such as heat, can lead to protection from other stressors through cyto-protective memory present in the activation of protective signaling [58, 59, 85]. It is possible that the underlying purpose of the selection seen in the populations are involved in histone and transcriptional modifications leading to cross-tolerance brought about by numerous small adaptions to a challenging environment. The regulation of these types of signaling processes (i.e. *MAPK, NF-κB*) and the initiation of regulatory kinases and genes (*PRKCA, CDKN3, ADM, OFLM4*) that assist in activating processes that protect cells from oxidative damage. Selective pressure on *PRKCA* also indicates the presence of prolactin signaling, shown to be a heat stress related signaling system in cattle. There is also evidence of selective pressure for protection of cardiovascular health under metabolic stress. In the sampling populations of Uganda and Rwanda there was some indication of adaption to environmental challenges from heat present in the some of the observable phenotypes of the birds. Heat stress related phenotypes such as frizzled and Naked Neck were observed within the sampling populations.

## Conclusion

The three populations displayed evidence of stratification, which indicated admixture among the populations and especially among the ecotypes within a country. The strong selection signal in all populations at *PRKCA*, as well as, the population-specific selection signals for *CDKN3, DLC1,* and *ADM,* strongly indicates that these populations have developed means to maintain cellular homeostasis despite the presence of oxidative and metabolic stress. Our results indicate that the birds may use calcium-mediated responses to counteract environmentally generated oxidative stress. Results from the pairwise comparison, along with the over-representation of GO terms related to stress responses, also support this notion. The populations shared multiple statistically significant GO terms and genes related to selection pressure on kinases and calcium activity. Overall, this evidence of selective pressure on genes related to kinases, calcium activity, and oxidative stress responses provides a window through which to discern mechanisms used by chickens to tolerate the effects of a challenging environment.

## Additional files

Additional file 1: Table S1. Table showing family, individual, and sex identification for study samples. (XLSX 37 kb) Additional file 2: Table S2. Locations and relations of shared genes and statistically significant SNPs for iHS analysis. (DOCX 15 kb) Additional file 3: Table S3. Locations and relations of unique genes and statistically significant SNPs for iHS analysis. (DOCX 16 kb) Additional file 4: Figure of F_st _sliding window analysis showing selection pressure around *PRKCA*. (PDF 10746 kb) Additional file 5: Figure showing regions where Ugandan and Rwandan chickens were sampled. (PDF 4238 kb)
