# Supplementary material for: Genomic analysis of Ugandan and Rwandan chicken ecotypes using a 600 k genotyping array
Source: BMC Genomics. 2016 May 26;17:407. doi: 10.1186/s12864-016-2711-5 (PMC4882793; doi:10.1186/s12864-016-2711-5)
Supplement: Additional file 2: Table S2. — Locations and relations of shared genes and statistically significant SNPs for iHS analysis. (DOCX 15 kb) [file 12864_2016_2711_MOESM2_ESM.docx]

Additional file 2: Table S1. Locations and relations of shared genes and statistically significant SNPs for iHS analysis

| Group | Probe ID | Chr | Relationship | Position | Gene Distance | Gene | \|iHS\| |
| --- | --- | --- | --- | --- | --- | --- | --- |
| Uganda | AX-75908503 | 18 | Intron | 7674793 | 0 | PRKCA | 3.95 |
| Uganda | AX-75908504 | 18 | Intron | 7675326 | 0 | PRKCA | 5.20 |
| Uganda | AX-75908506 | 18 | Intron | 7676016 | 0 | PRKCA | 5.03 |
| Uganda | AX-75908507 | 18 | Intron | 7676721 | 0 | PRKCA | 4.02 |
| Uganda | AX-75908514 | 18 | Intron | 7678101 | 0 | PRKCA | 5.19 |
| Uganda | AX-75908534 | 18 | Intron | 7682388 | 0 | PRKCA | 4.10 |
| Rwanda | AX-75908503 | 18 | Intron | 7674793 | 0 | PRKCA | 3.86 |
| Rwanda | AX-75908504 | 18 | Intron | 7675326 | 0 | PRKCA | 4.17 |
| Rwanda | AX-75908506 | 18 | Intron | 7676016 | 0 | PRKCA | 3.80 |
| Rwanda | AX-75908514 | 18 | Intron | 7678101 | 0 | PRKCA | 4.22 |
| Kuroiler | AX-80781124 | 18 | Intron | 7661265 | 0 | PRKCA | 3.98 |
| Kuroiler | AX-75908469 | 18 | Intron | 7664399 | 0 | PRKCA | 3.89 |
| Kuroiler | AX-75908470 | 18 | Intron | 7664918 | 0 | PRKCA | 5.40 |
| Kuroiler | AX-75908474 | 18 | Intron | 7665324 | 0 | PRKCA | 4.26 |
| Kuroiler | AX-75908477 | 18 | Intron | 7666703 | 0 | PRKCA | 4.26 |
| Kuroiler | AX-75908480 | 18 | Intron | 7667210 | 0 | PRKCA | 4.36 |
| Kuroiler | AX-75908482 | 18 | Intron | 7668351 | 0 | PRKCA | 3.45 |
| Kuroiler | AX-75908484 | 18 | Intron | 7669186 | 0 | PRKCA | 3.45 |
| Kuroiler | AX-75908494 | 18 | Intron | 7671031 | 0 | PRKCA | 4.96 |
| Kuroiler | AX-75908498 | 18 | Intron | 7672436 | 0 | PRKCA | 3.47 |
| Kuroiler | AX-75908502 | 18 | Intron | 7674562 | 0 | PRKCA | 4.45 |
| Kuroiler | AX-75908503 | 18 | Intron | 7674793 | 0 | PRKCA | 5.29 |
| Kuroiler | AX-75908515 | 18 | Intron | 7678145 | 0 | PRKCA | 3.88 |
| Kuroiler | AX-75908519 | 18 | Intron | 7678802 | 0 | PRKCA | 3.33 |
