# Supplementary material for: Genomic analysis of Ugandan and Rwandan chicken ecotypes using a 600 k genotyping array
Source: BMC Genomics. 2016 May 26;17:407. doi: 10.1186/s12864-016-2711-5 (PMC4882793; doi:10.1186/s12864-016-2711-5)
Supplement: Additional file 3: Table S3. — Locations and relations of unique genes and statistically significant SNPs for iHS analysis. (DOCX 16 kb) [file 12864_2016_2711_MOESM3_ESM.docx]

Additional file 3: Table S2. Locations and relations of unique genes and statistically significant SNPs for iHS analysis.

| Group | Probe ID | Chr | Relationship | Position | Gene Distance | Gene | \|iHS\| |
| --- | --- | --- | --- | --- | --- | --- | --- |
| Rwanda | AX-76707891 | 4 | Intron | 64044265 | 0 | DLC1 | 3.84 |
| Rwanda | AX-76707900 | 4 | Intron | 64049731 | 0 | DLC1 | 3.94 |
| Rwanda | AX-76707902 | 4 | Intron | 64051758 | 0 | DLC1 | 4.18 |
| Rwanda | AX-76707906 | 4 | Intron | 64052556 | 0 | DLC1 | 4.29 |
| Rwanda | AX-76707918 | 4 | Intron | 64064081 | 0 | DLC1 | 3.36 |
| Rwanda | AX-76707933 | 4 | Intron | 64064081 | 0 | DLC1 | 3.50 |
| Uganda | AX-76877064 | 5 | Upstream | 57187445 | 817111 | CDKN3 | 3.99 |
| Uganda | AX-76877067 | 5 | Upstream | 57189726 | 819392 | CDKN3 | 4.73 |
| Uganda | AX-76877069 | 5 | Upstream | 57190070 | 819736 | CDKN3 | 4.32 |
| Uganda | AX-76877082 | 5 | Upstream | 57195249 | 824915 | CDKN3 | 3.99 |
| Uganda | AX-76877085 | 5 | Upstream | 57196471 | 826137 | CDKN3 | 4.62 |
| Uganda | AX-76877089 | 5 | Upstream | 57197820 | 827486 | CDKN3 | 4.58 |
| Uganda | AX-76877096 | 5 | Upstream | 57199367 | 829033 | CDKN3 | 4.16 |
| Uganda | AX-76877106 | 5 | Upstream | 57202790 | 832456 | CDKN3 | 4.82 |
| Uganda | AX-76877125 | 5 | Upstream | 57211269 | 840935 | CDKN3 | 3.92 |
| Uganda | AX-76877127 | 5 | Upstream | 57211684 | 841350 | CDKN3 | 4.26 |
| Uganda | AX-76877132 | 5 | Upstream | 57214099 | 843765 | CDKN3 | 3.92 |
| Kuroiler | AX-76888764 | 5 | Downstream | 8390939 | 32222 | ADM | 4.21 |
| Kuroiler | AX-76888768 | 5 | Downstream | 8393005 | 30156 | ADM | 4.27 |
| Kuroiler | AX-76888769 | 5 | Downstream | 8393479 | 29682 | ADM | 3.54 |
| Kuroiler | AX-76888771 | 5 | Downstream | 8394252 | 28909 | ADM | 3.91 |
| Kuroiler | AX-76888774 | 5 | Downstream | 8395018 | 28143 | ADM | 4.03 |
| Kuroiler | AX-76888778 | 5 | Downstream | 8397550 | 25611 | ADM | 4.71 |
| Kuroiler | AX-76888780 | 5 | Downstream | 8398497 | 24664 | ADM | 5.80 |
| Kuroiler | AX-76888781 | 5 | Downstream | 8399379 | 23782 | ADM | 3.48 |
| Kuroiler | AX-76888784 | 5 | Downstream | 8400321 | 23782 | ADM | 4.79 |
| Kuroiler | AX-76888787 | 5 | Downstream | 8401224 | 21937 | ADM | 3.72 |
| Kuroiler | AX-76888823 | 5 | Downstream | 8413517 | 9644 | ADM | 4.00 |
| Kuroiler | AX-76888852 | 5 | Upstream | 8426005 | 208 | ADM | 3.67 |
