# Supplementary material for: Genomic analysis of Ugandan and Rwandan chicken ecotypes using a 600 k genotyping array
Source: BMC Genomics. 2016 May 26;17:407. doi: 10.1186/s12864-016-2711-5 (PMC4882793; doi:10.1186/s12864-016-2711-5)
Supplement: Additional file 4: — Figure of Fst sliding window analysis showing selection pressure around PRKCA. (PDF 10746 kb) [file 12864_2016_2711_MOESM4_ESM.pdf]

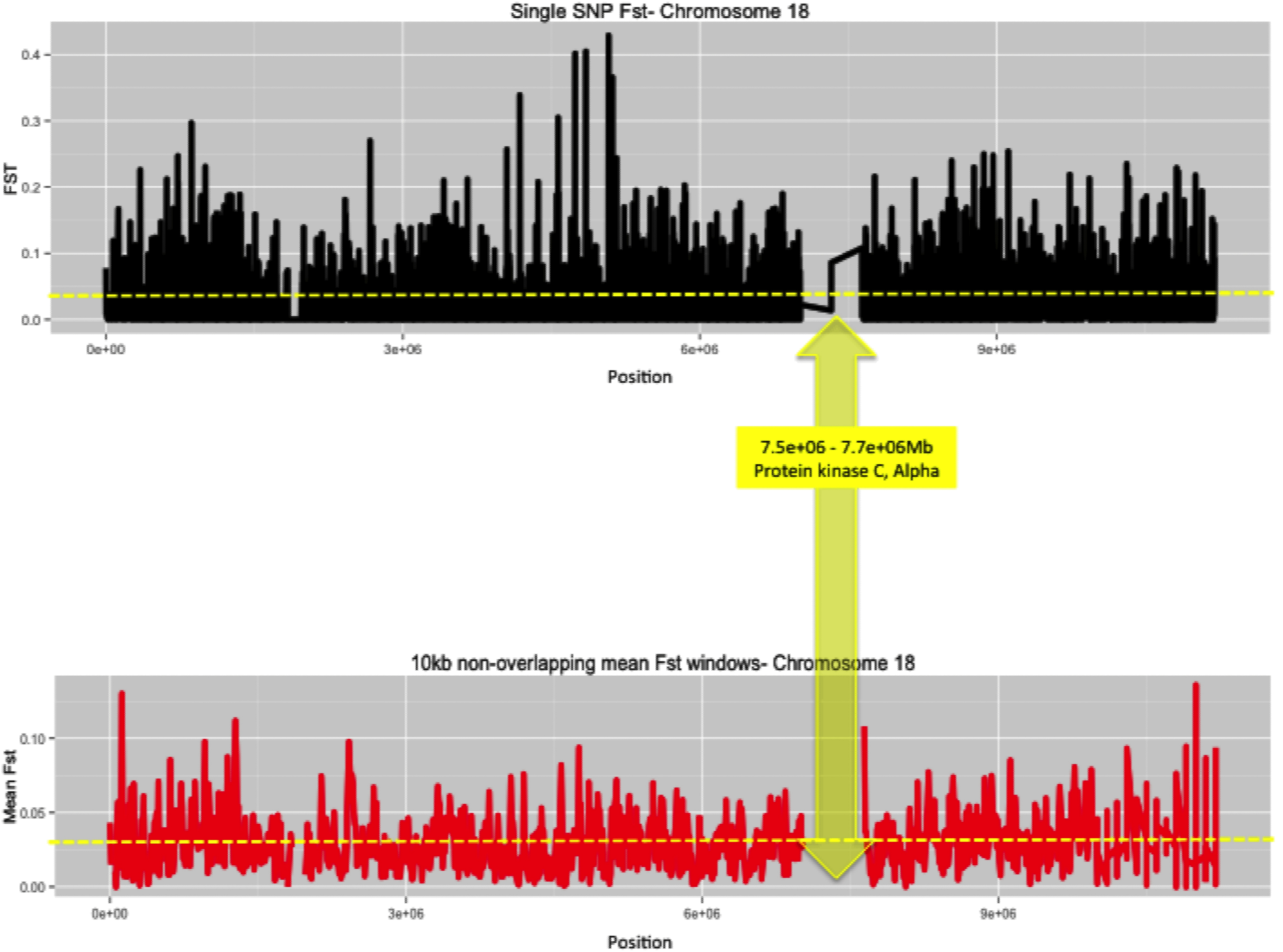

Single SNP Fst- Chromosome 18
FST
0.0
0.1
0.2
0.3
0.4
0e+00
3e+06
6e+06
9e+06
Position
7.5e+06 - 7.7e+06Mb
Protein kinase C, Alpha
10kb non-overlapping mean Fst windows- Chromosome 18
Mean Fst
0.00
0.05
0.10
0e+00
3e+06
6e+06
9e+06
Position
