# Supplementary figures and images for: Genomic analysis of Ugandan and Rwandan chicken ecotypes using a 600 k genotyping array
Source: BMC Genomics. 2016 May 26;17:407. doi: 10.1186/s12864-016-2711-5 (PMC4882793; doi:10.1186/s12864-016-2711-5)

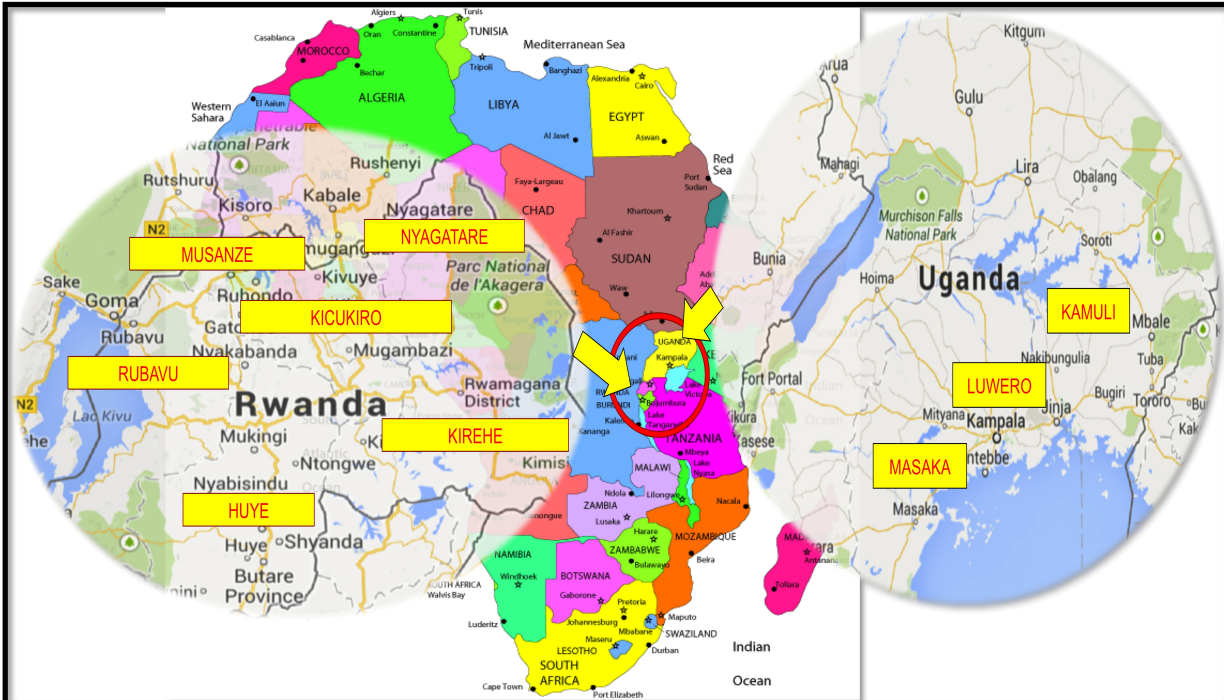

Supplement: Additional file 5: — Figure showing regions where Ugandan and Rwandan chickens were sampled. (PDF 4238 kb) [file 12864_2016_2711_MOESM5_ESM.pdf]
